# Chronic Remote Ischemic Conditioning May Mimic Regular Exercise:Perspective from Clinical Studies

**DOI:** 10.14336/AD.2017.1015

**Published:** 2018-02-01

**Authors:** Wenbo Zhao, Sijie Li, Changhong Ren, Ran Meng, Xunming Ji

**Affiliations:** ¹Department of Neurology, Xuanwu Hospital, Capital Medical University, Beijing, China; ^2^Beijing Key Laboratory of Hypoxic Conditioning Translational Medicine, Xuanwu Hospital, Capital Medical University, Beijing, China; ^3^Beijing Municipal Geriatric Medical Research Center, Beijing, China; ^4^National Clinical Research Center for Geriatric Disorders, Beijing, China

**Keywords:** chronic remote ischemic conditioning, regular exercise, organ protection

## Abstract

Chronic remote ischemic conditioning (RIC), particularly long-term repeated RIC, has been applied in clinical trials with the expectation that it could play its protective roles for protracted periods. In sports medicine, chronic RIC has also been demonstrated to improve exercise performance, akin to improvements seen with regular exercise training. Therefore, chronic RIC may mimic regular exercise, and they may have similar underlying mechanisms. In this study, we explored the common underlying mechanisms of chronic RIC and physical exercise in protecting multiple organs and benefiting various populations, the advantages of chronic RIC, and the challenges for its popularization. Intriguingly, several underlying mechanisms of RIC and exercise have been shown to overlap. These include the production of many autacoids, enhanced ability for antioxidant activity, modulating immune and inflammatory responses. Therefore, it appears that chronic RIC, just like regular exercise, has beneficial effects in unhealthy, sub-healthy and healthy individuals. Compared with regular exercise, chronic RIC has several advantages, which may provide novel insights into the area of exercise and health. Chronic RIC may enrich the modes of exercise, and benefit individuals with severe diseases. Also, the disabled, and sub-healthy individuals are likely to benefit from chronic RIC either as an alternative to exercise or an adjunct to pharmacological or non-pharmacological therapy.

## 1. Regular exercise

### Benefits of exercise

An exercise is a form of physical activity that is planned, structured, repetitive, and purposeful with the primary objective to improve or maintain one or more organs of physical fitness. To date, studies have demonstrated the benefits of regular exercise in reducing risks of mortality occurring from all causes. A strong inverse relationship has also been observed between exercise and risks for coronary disease, adverse cardiac events, and cardiovascular death [1]. Furthermore, exercise may protect against many cancers (e.g., breast, intestinal, prostate, and endometrial cancers) [2-4], and reduce psychiatric disorders (e.g., stress, anxiety, and depression) [5]. Further, aerobic exercise and resistance training have been demonstrated to be associated with weight loss and reduction in body fat, which may lead to significant health benefits over the course of a lifetime [6].

The underlying mechanisms of exercise-induced protection include increased production of heat shock protein 72 [7], modulation of the nitric oxide (NO) pathway [8], increased antioxidant capacity [9] and adenosine triphosphate-sensitive potassium (K_ATP_) channels function [10], and activation of the opioid system [11]. Moreover, the protection induced by exercise has two phases of protection. The initial phase of protection begins 0.5 hours after exercise and lasts for 3 hours, and the late phase of protection, which is far more robust than the first protection, is achieved 24 hours post-exercise and continues for over nine days [12].

### Dilemmas of exercise

However, things are never as simple as we thought. Inappropriate exercise may cause injury or damage to our body. The most common risk of exercise is musculoskeletal injury [13], and other more serious risks include arrhythmia, sudden cardiac arrest [14], myocardial infarction [14], dehydration, and heat-related risks ranging from mild fatigue to death [15]. Besides, some people may not be able to do exercise because of disabilities, history of pulmonary or cardiac diseases, or orthopedic history [16]. More importantly, with the increasingly fierce competition in modern society, a large number of people have subpar health because they fail to do regular exercise with an excuse of having no time [17]. Therefore, surrogates to exercise that are safer, time-saving, easier to popularize, and have the benefits of exercise, are urgently needed.

**Table 1:** Comparison of mechanisms between RIC and exercise.

	Different mechanisms	Common mechanisms
RIC	Neuropathway Increased cyclooxygenase-2 Elevated endoplasmic reticulum stress proteins	Increased HSP Increased NO Improve KATP function Increased antioxidant capacity Induced autophagy Involvement of opioid system Regulation of Immune/inflammatory system
Regular exercise		

RIC, remote ischemic conditioning; HSP, heat shock protein; NO, nitric oxide; K_ATP_, adenosine triphosphate-sensitive potassium.

## 2. Ischemic conditioning

### From bench to bedside

Thirty years ago, Murry and colleagues first discovered the phenomenon of ischemic pre-conditioning in an animal experiment [18]. The seminal discovery that brief episodes of ischemia followed by reperfusion could significantly reduce myocardial infarct size gave rise to the area of myocardial protection firstly and then propagated to multi-organ protection (e.g., liver, nervous system, kidney, skeletal muscle, etc.) [19-21, 18]. Ischemic preconditioning has evolved into remote ischemic conditioning (RIC), which includes pre-conditioning, per-conditioning, and post-conditioning [22]. Although the underlying mechanisms of RIC are still unclear, it was found to be safe and well tolerated in both patients and healthy volunteers[23, 24]. Three pathways have been proposed (i.e., humoral pathway, neural pathway, and immunological pathway) [25, 26]. Furthermore, like exercise, RIC has been shown to induce two distinct phases of organ protection. The early phase occurs immediately after RIC stimulus and lasts 2 to 3 hours, while the later phase follows 12 to 24 hours later and lasts 48 to 72 hours [27, 28]

### Chronic RIC

The use of long-term repeated RIC comes with the expectation that RIC can play its protective roles consistently; this RIC treatment protocol is now called chronic RIC. Clinical studies have demonstrated that chronic RIC could reduce adverse clinical events and improve neurological function [29-31], which was rare in previous studies using a once-only RIC treatment protocol. This discrepancy may be attributed to the idea that different organs respond to RIC stimulus differently and various studies use different endpoints, but the difference in RIC treatment protocols may be a more plausible explanation. A once-only RIC can exert its protective roles for 72 hours at most with an unprotected period lasting 10-20 hours [32]. Hence, if RIC is performed once or twice daily for an extended period, it can exert its protective effects continuously, and the two distinct phases of organ protection can play their roles simultaneously or successively.

### Chronic RIC in Sport Medicine

Intriguingly, some sports specialists, inspired by the favorable effects of RIC on skeletal muscles and endothelial function [33, 34], applied RIC to exercise training, as intense exercise has been demonstrated to lead to a form of cardiac and skeletal muscles ischemic insult [35]. To date, RIC has been shown to improve the maximal performance in highly trained swimmers [36], enhance 5-km time trial performance and attenuate the submaximal level of blood lactate during the incremental running test [37]. Similarly, swimming performance can be improved by regular pool training for several weeks [38], and incorporation of taper for 10 to 14 days can significantly shorten 5-km running time [39]. Therefore, RIC could improve exercise performance as does regular interval exercise training.

## 3. Common mechanisms of chronic RIC and regular exercise

Given the similar time window of early and late phase protection seen with both exercise and RIC, and their comparable effects on improving exercise performance, it is reasonable to speculate that the underlying mechanisms of RIC likely overlap with those of exercise. As the mechanisms of RIC and exercise in cardio-protection have been well investigated, here we summarize several central common mechanisms of RIC and exercise mainly in the field of cardio-protection (Table 1).

### Increased heat shock protein

Heat shock protein (HSP) 70 family, especially HSP72, has been demonstrated to be associated with cardioprotection. Previous studies found significantly increased HSP72 levels after acute aerobic exercise, associated with protection against myocardial ischemia/reperfusion injury [40, 41]. Similarly, increased HSP72 has been reported after RIC stimulus, which likely plays an important role in RIC-induced cardioprotection [42]. Therefore, the overexpression of HSP72 may be one of the underlying common pathways of RIC and exercise in cardioprotection.

### Involvement of the NO pathway

Previous studies have found that during myocardial ischemia/reperfusion, increased NO levels could reduce mitochondrial reactive oxygen species (ROS) production and increase S-nitrosylation of cardiac proteins, which alleviated ischemic/reperfusion injurious processes (e.g., apoptosis) [43, 44]. Studies have demonstrated that exercise promoted endothelial NO synthase activity, increased the production of NO and improved endothelial cell function [45]. Similarly, chronic RIC has been demonstrated to significantly improve flow-mediated dilation and enhance endothelial NO synthase expression in patients with coronary heart disease [46].

### Adenosine triphosphate-sensitive potassium

Studies showed that exercise could increase the expression of the K_ATP_ channel in distant vital organs, which may improve its metabolic-sensing capability and help organs better maintain active status during ischemia [47, 48]. Intriguingly, RIC triggers intracellular kinase signaling that induces the opening of K_ATP_ channel, which prevents the formation of mitochondrial permeability transition pore (MPTP) [49, 50]. Therefore, chronic RIC’s effects on K_ATP_ may be one of the reasons why it has a protective impact on multiple organs just like regular exercise.

### Increased antioxidant capacity

Oxidative stress plays an essential role in the process of many diseases, and ROS produced by mitochondria contribute to cell necrosis and apoptosis. However, the human body has a complex antioxidant system, which includes superoxide dismutase (SOD), glutathione peroxidase, and catalase [51]. Studies have demonstrated that exercise can increase catalase and glutathione peroxidase levels, and also promote SOD activity [52, 12, 53]. Furthermore, exercise leads to a transient release of ROS, which may increase the antioxidant capacity of the human body [52]. Similarly, RIC has been demonstrated to enhance the scavenging of ROS and increase endogenous antioxidant activity in ischemia/reperfusion animal [54, 55].

### Induced autophagy

Autophagy is a process for eliminating dysfunctional organelles and protein aggregates, which is a kind of endogenous protection and required for cellular survival and homeostasis in response to stress. Studies have found that protective effects of RIC on cell survival were mediated by autophagy pathway, and autophagy participants in RIC-induced protection [56, 57]. Exercise induces the adaptational response from multiple organs (primarily in skeletal muscle), which will benefit human body [58, 59]. Recently, autophagy has been found to be an essential process involved in conserving and recycling the cellular resources, an important process of the adaptation response [60].

### Involvement of opioid system

In 1995, Schultz et al. found that RIC-induced endogenous opioid peptide had cardioprotective effects on reducing myocardial ischemic injury [61]. This autacoid binds to its respective receptors on the cytomembrane to stimulate several intracellular signaling pathways. Similarly, studies have demonstrated significant increase in beta-endorphin (an endogenous opioid) levels after acute bouts of running exercise. And exercise has also been shown to promote the expression of opioid receptors. Therefore, the opioid system plays important roles in both RIC- and exercise-induced protections.

### Regulation of immune/inflammatory system

Exercise has direct beneficial effects on the cellular immune system, and it can mobilize NK and T cells to circulation during exercise [62]. The immune cell activity may also be influenced by the exercise-induced release of immune regulatory cytokines [63]. In addition, numerous studies have demonstrated that both acute and long-term exercise can regulate the circulating monocytes, which play important roles in both acute and chronic inflammation [64]. Similarly, RIC has also been demonstrated to influence inflammatory response and immune cells, and these are the essential underlying mechanisms of RIC-induced protection. Konstantinov et al. showed that RIC reduces inflammatory gene expression with 15 minutes and 24 hours after RIC stimulus [65]. Furthermore, RIC stimulus dramatically changes the immune response, which induces protection [66].

## 4. Advantages and challenges

From the data available so far, it appears that chronic RIC mimics regular exercise. Further studies are however urgently needed to validate this phenomenon. Compared with regular exercise, chronic RIC may have the following advantages. It is much safer and more convenient, children and the elderly can use it without concerns of getting hurt or suffer from severe adverse effects [30]. Even those individuals, who are unable to do exercise (e.g., the disabled or those encumbered with several debilitating diseases), are likely to benefit from chronic RIC either as an alternative or an adjunct to pharmacological or non-pharmacological therapy. More importantly, this approach would free up additional time for leisurely activities such as watching TV, browsing the Internet, having meetings or even extending the professional working activities. Therefore, even office workers, who are at risk for below-par health or already suffering from below-par health, can incorporate RIC procedure regularly. Besides, RIC can be used in patients with severe acute diseases (e.g., acute myocardial infarction, acute stroke) where RIC can play its protective roles immediately [67, 68].

However, there are still several hurdles for popularizing chronic RIC. Currently, different chronic RIC protocols are used in various studies, the periods of using chronic RIC vary from 1 week to 1 year, and its frequencies vary from twice daily to once every two weeks. Although chronic RIC benefits health and protects organs from injury, its optimal protocol is still unclear. Most importantly, our group found that young people do not wish to commit to chronic RIC because it is inconvenient to be carried around, whereas elderly patients insist on performing RIC regularly. Therefore, a more portable device could improve the compliance of young people and people with high mobility.

## 5. Future investigations

Currently, it is well established that exercise has widespread health benefits by positively affecting nearly all organ systems of the body [5, 1-3, 6, 4]. The benefits include reduced mortality from all causes, weight control, prevention of cancers, increased longevity, improved mental health, and anti-hypertension effects. Chronic RIC has been tested extensively in patients with cerebrovascular diseases, cardiovascular diseases, renal diseases, respiratory diseases and cancer [69, 70]. Moreover, chronic RIC has been demonstrated to have sustained blood pressure lowering effect in normotensive or prehypertensive subjects [71, 72]. However, there is still no evidence supporting that chronic RIC has beneficial effects on body weight, cancer prevention, mental health [73] or lifespan extension. As chronic RIC and regular exercise share similar underlying mechanisms, it is likely that chronic RIC has widespread health benefits as does regular exercise. Therefore, it is reasonable to further investigate the beneficial effects of chronic RIC in non-patients (e.g., office workers with subpar health). In the future, besides examining the beneficial effects of chronic RIC in various patients, efforts should also be made to investigate the impact of chronic RIC in healthy or and in people with subpar health.

## 6. Conclusion

Chronic RIC-mediated beneficial effects have similar underlying mechanisms as exercise-related favorable effects. Based on these similarities, we speculate that chronic RIC likely has widespread health benefits as regular exercise. Nonetheless, it remains to be determined whether chronic RIC has beneficial effects on body weight loss, cancer prevention, mental health, and lifespan extension. Furthermore, before the widespread application of chronic RIC, systematic and rigorous studies examining both beneficial and adverse effects of various RIC protocols and devices are required.
